# Integrated bioinformatics to identify potential key biomarkers for COVID-19-related chronic urticaria

**DOI:** 10.3389/fimmu.2022.1054445

**Published:** 2022-12-01

**Authors:** Teng Zhang, Hao Feng, Xiaoyan Zou, Shixiong Peng

**Affiliations:** ^1^ Department of Dermatology, Changsha Hospital of Traditional Chinese Medicine (Changsha Eighth Hospital), Changsha, China; ^2^ Department of Dermatology, Hunan Provincial People’s Hospital, Changsha, China; ^3^ Department of Dermatology, Maternal and Child Health Hospital of Hubei Province, Tongji Medical College, Huazhong University of Science and Technology, Wuhan, China

**Keywords:** chronic Urticaria (CU), COVID-19, bioinformatics, biomarker, immunology

## Abstract

**Background:**

A lot of studies have revealed that chronic urticaria (CU) is closely linked with COVID-19. However, there is a lack of further study at the gene level. This research is aimed to investigate the molecular mechanism of COVID-19-related CU *via* bioinformatic ways.

**Methods:**

The RNA expression profile datasets of CU (GSE72540) and COVID-19 (GSE164805) were used for the training data and GSE57178 for the verification data. After recognizing the shared differently expressed genes (DEGs) of COVID-19 and CU, genes enrichment, WGCNA, PPI network, and immune infiltration analyses were performed. In addition, machine learning LASSO regression was employed to identify key genes from hub genes. Finally, the networks, gene-TF-miRNA-lncRNA, and drug-gene, of key genes were constructed, and RNA expression analysis was utilized for verification.

**Results:**

We recognized 322 shared DEGs, and the functional analyses displayed that they mainly participated in immunomodulation of COVID-19-related CU. 9 hub genes (CD86, FCGR3A, AIF1, CD163, CCL4, TNF, CYBB, MMP9, and CCL3) were explored through the WGCNA and PPI network. Moreover, FCGR3A, TNF, and CCL3 were further identified as key genes *via* LASSO regression analysis, and the ROC curves confirmed the dependability of their diagnostic value. Furthermore, our results showed that the key genes were significantly associated with the primary infiltration cells of CU and COVID-19, such as mast cells and macrophages M0. In addition, the key gene-TF-miRNA-lncRNA network was constructed, which contained 46 regulation axes. And most lncRNAs of the network were proved to be a significant expression in CU. Finally, the key gene-drug interaction network, including 84 possible therapeutical medicines, was developed, and their protein-protein docking might make this prediction more feasible.

**Conclusions:**

To sum up, FCGR3A, TNF, and CCL3 might be potential biomarkers for COVID-19-related CU, and the common pathways and related molecules we explored in this study might provide new ideas for further mechanistic research.

## Introduction

Chronic urticaria (CU), as one of the most common chronic pruritus and immunological skin diseases in dermatology, and it is manifested as wheal or angioedema occurring for more than six weeks (1). Epidemiological studies reported that the global prevalence of CU is about 1.4%, which has been rising in the context of the COVID-19 pandemic (2, 3). Notably, CU exerts a great impact on the life quality of adults, pediatric patients, and their families. Recurrent pruritus and rash make CU patients vulnerable to interruptions in work and daily life activities and sleep disturbances (4). Furthermore, it has become a burden on the utilization of health care resources and increased global costs (5). However, the pathogenesis of CU has not been completely understood, and there are few objective biomarkers. The diagnosis of CU is primarily dependent on symptoms and the history of the illness. Since it can be self-limited, patients’ symptoms may disappear when they see a doctor. Thus, the disease could not get an accurate assessment. Therefore, it is of great significance to further explore the pathogenesis and identify key biomarkers of CU.

The etiology of CU is very complicated and not completely disclosed. It is currently considered that the central pathogenesis of CU is the degranulation of the mast cells activated by diverse causes (6). For example, long-term exogenous physical stimulation (such as pressure) can induce the occurrence of urticaria (7). In addition, the endogenous causes of CU may be more widespread, including chronic autoimmune diseases and insidious chronic infections (e.g. as hepatitis virus, and human herpesvirus-6) (8). According to the latest studies found that COVID-19 could be associated with various immune diseases, since SARS-CoV-2 causes a violent inflammatory response and releases large amounts of cytokines (9). Notably, several studies of COVID-19 hospitalization patients have indicated that approximately 39.5% of patients present with different allergic diseases, and the incidence of CU is 10%. Moreover, CU is one of the skin disorders most severely affected by COVID-19, and it severely impairs CU patient care (10, 11). Besides, what has caused much concern is the adverse event in which CU occurs secondary to the COVID-19 vaccine (12, 13). These suggest that COVID-19 and CU are closely related, but hardly any further genetic research exists.

With the advancement of microarray technology, bioinformatics, an interdisciplinary method, can enable researchers to reveal the nosogenesis of the disease more thoroughly from genetics (14). In the present research, we applied the integrated bioinformatic approach to investigate immune cells infiltration, reveal molecular regulatory networks, and identify the shared key genes involved in the pathogenesis of CU and COVID-19, which might provide new perspectives for the biological mechanisms of COVID-19-related CU.

## Material and method

### Datasets preparation

**Figure 1:**
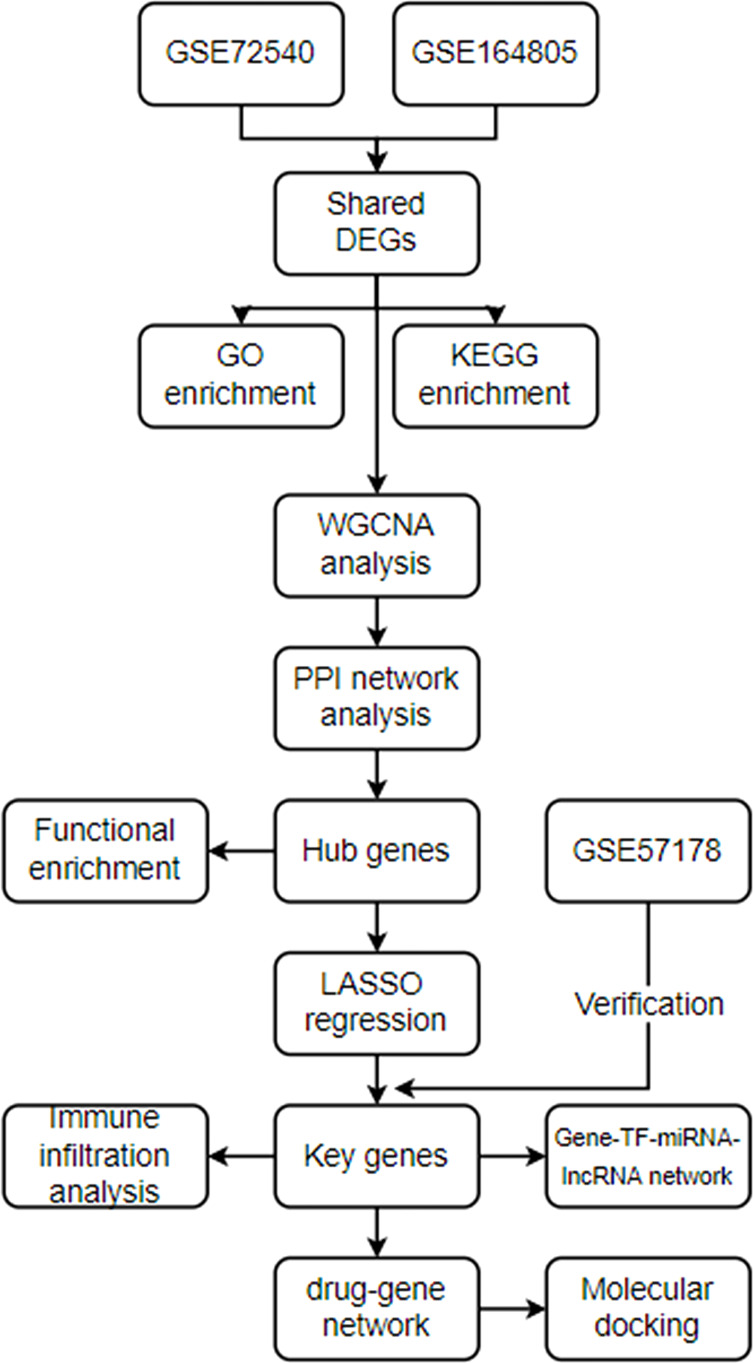
The analysis processes.

The analysis processes of this research are displayed in **Figure 1**. GSE72540, GSE164805, and GSE57178 were downloaded from the GEO website (https://www.ncbi.nlm.nih.gov/geo/ ) (**Table 1**). GSE164805 consisted of 10 COVID-19 patients and 5 controls. We selected ten CU lesional skin tissues and eight controls from 31 samples’ RNA expression profiling of GSE72540, as well as six CU lesional skin tissues and seven controls from 18 samples of GSE57178.

**Table 1:** Details of the RNA expression profile datasets.

Dataset	Platform	PMID	Whole samples (selected subjects, CU or COVID-19/Control)	Sample type	Gender	Age
					(female/male)	(mean ± SD)
GSE72540	GPL16699	28407332	31 (18,10/8)	Skin tissue	19/11	43.5 ± 17.38
	Agilent-085982 Arraystar human lncRNA V5 microarray					
GSE164805	GPL6244	33679778	15 (15,10/5)	PBMCs	2/13	56.4 ± 7.54
	Agilent-039494 SurePrint G3 Human GE v2 8x60K Microarray 039381 (Feature Number version)					
GSE57178	GPL26963	26302730	18 (13,6/7)	Skin tissue	11/3	43.5 ± 12.95
	[HuGene-1_0-st] Affymetrix Human Gene 1.0 ST Array [transcript (gene) version]					

### Identifying shared differently expressed genes between COVID-19 and CU

The GEO2R tool, an online interactive tool to identify DEGs by comparing two datasets in the GEO series (15), was used to normalize, preprocess the data, and identify DEGs among the patient sample and control. P-value <0.05 and |log_2_ FC| >1 were considered as the DEGs. The common genes between COVID-19 and CU were identified as the shared DEGs.

### Genes enrichment analysis

Gene ontology (GO) annotation analysis (biological process, cellular component, and molecular function) and KEGG pathway enrichment analysis were executed *via* R’s cluster profile package (16), and P-value <0.05 was the cut-off.

### Weighted gene co-expression network analysis analysis

The shared DEGs were performed further analyzed by R package “WGCNA” to explore the gene modules significantly related to disease (17). In a word, outlier samples were excluded by the hierarchical clustering analysis at first. Next, the “pickSoftThreshold” in the WGCNA package was adopted to select the appropriate soft powers β (soft power = 2). An adjacency matrix was generated and then converted to a topological overlap matrix (TOM). Based on the differential TOM measures, the genes of similar expression patterns were classified into different modules *via* average linkage hierarchical clustering. Finally, the relevance between the modules and clinical features was computed.

### Protein-protein interaction network analysis

The STRING tool, a database for customizing protein-protein networks and finding functional characterization of gene sets (18), was used to explore protein-protein interaction (PPI) networks of the correlated modules genes of WGCNA, and it was visualized *via* the Cytoscape. Furthermore, the module analysis of the PPI network was performed using MCODE plug-in.

### Identification and enrichment analysis of hub genes

The hub gene was identified through four algorithms (maximal clique centrality (MCC), maximum neighborhood component (MNC), Degree, and edge percolated component) (EPC)) of the cytoHubba plug-in of Cytoscape (19). Further genes enrichment analysis of hub genes was accomplished *via* ClueGO plug-in.

### Recognition of key genes through machine learning and ROC curve analyses

In order to further filter candidate genes for CU diagnosis, we used a machine learning algorithm to perform LASSO regression. In short, we integrated clinical features and gene expression data to conduct LASSO regression analysis *via* the “glmnet” R package (20). ROC curve was applied to analyze the robustness of the diagnosis of the key genes. Besides, the GSE57178 was used for the validation set.

### Immune infiltration analysis

The immune infiltration analysis was performed by using CIBERSORT tool, a deconvolution algorithm by evaluating the expression of related genes based on gene expression (21), to calculate the ratio of twenty-two infiltrating lymphocyte subsets in CU and COVID-19 samples. The correlations between each immune cell and among immune cells and hub genes were calculated using GraphPad Prism (version 8.0.2) (22).

### Construction of gene- transcription factor -miRNA-lncRNA network of the key genes

In order to understand the molecular mechanism of disease, we constructed a gene-TF-miRNA-lncRNA network. Firstly, the TRRUST database, a manually curated database of human and mouse transcriptional regulatory networks (23), was first used to predict the TFs interacting with the key genes. Next, the tools (24, 25), online databases of prediction of RNA interactomes, including PITA, miRmap, microT, miRanda, PicTar, and TargetScan, were applied to explore the interaction of TF-miRNA. If it was recognized to be uniform in all the tools, it was considered valid. Then, the interaction of miRNA-lncRNA was identified *via* the databases (miRNet (26), starbase (27), and lncbasev3 (28)), and if it was recognized uniformity in all the tools, it was considered valid. The Cytoscape tool, an open source software platform for visualizing complex networks, was used for data visualization.

### Drug–Gene interaction and protein-protein docking analyses

The DGIdb tool (Drug-Gene Interaction database) (29) was used to investigate drug-gene interaction so as to identify drugs associated with the key genes. Besides, the interaction network was visualized *via* Cytoscape. Protein-protein docking was conducted by Cluspro 2.0 (https://cluspro.bu.edu/login.php ) (30), and the visualization of docked complexes was performed through the PyMOL software which was a cross-platform molecular graphics tool (31).

### Statistic analysis

The data of the two groups was analyzed with the unpaired Student’s t-test *via* the GraphPad Prism (version 8.0.2), and P-value <0.05 was considered to be statistically significant.

## Result

### Recognition of shared DEGs between COVID-19 and CU

**Figure 2:**
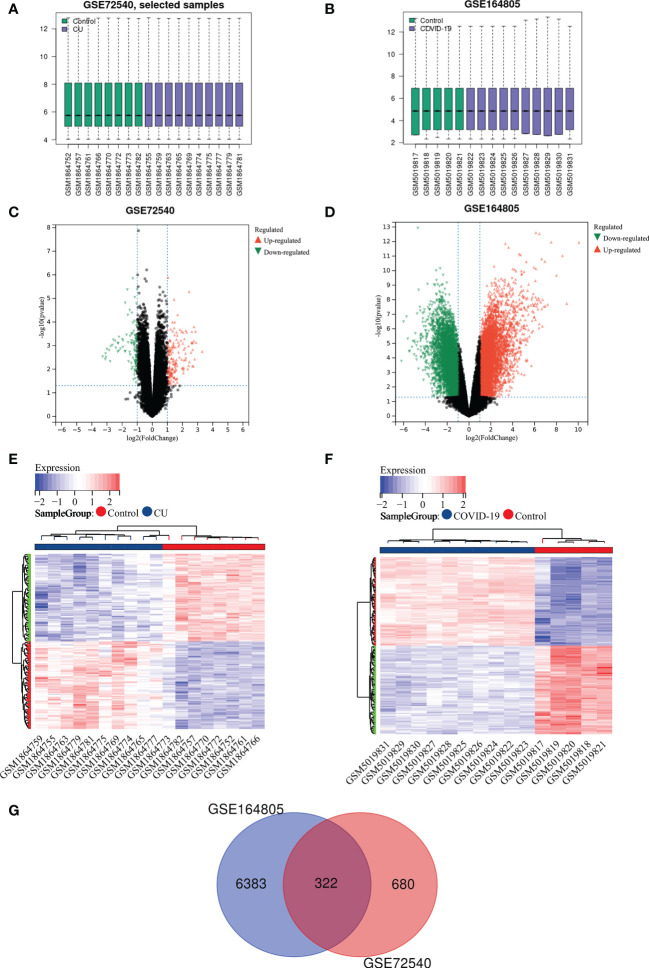
Recognition of shared DEGs between COVID-19 and CU. **(A)** Data standardization of GSE72540. **(B)** Data standardization of GSE164805. **(C)** The volcano plot of GSE72540. **(D)** The volcano map of GSE164805. **(E)** The heat map of the top 100 DEmRNAs of GSE72540. **(F)** The heat map of the top 100 DEmRNAs of GSE164805. **(G)** The shared DEGs between COVID-19 and CU by overlapping DEmRNAs.

**Figures 2A, B** show that the expression profile datasets, GSE72540 and GSE164805, were normalized, and their volcano plots are presented in **Figures 2C, D**. We identified 1033 differently expressed mRNAs (DEmRNAs) in GSE72540 and 6705 DEmRNAs in GSE164805. The heat maps of their top 100 DEmRNAs are shown in **Figures 2E, F**. We recognized 322 shared DEGs between CU and COVID-19 *via* integrated bioinformatics analysis in **Figure 2G** and **Table S1**.

### Function annotation analyses of the shared DEGs

**Figure 3:**
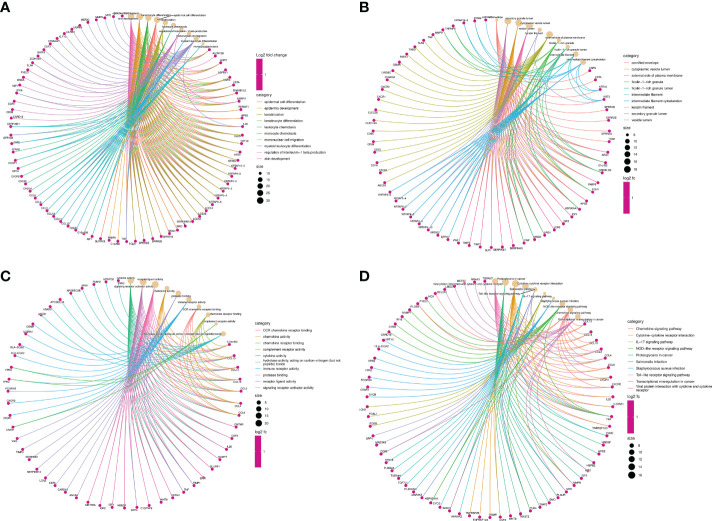
Function annotation analyses of the shared DEGs. **(A)** The top 10 items of GO biological process. **(B)** The top 10 items of GO cellular component. **(C)** The top 10 items of GO molecular function. **(D)** The top 10 signal pathways of KEGG.

The function annotation analyses of shared DEGs were performed to reveal the common biology functions between COVID-19 and CU (**Table 2**). As shown in **Figure 3A**, in the biological process, the main shared DEGs were involved into skin development, epidermal cell differentiation, leukocyte chemotaxis, mononuclear cell migration, etc. It can be seen from **Figure 3B** that in the cellular component, most of the shared DEGs participated in the cornified envelope, secretory granule lumen, cytoplasmic vesicle lumen, vesicle lumen, etc. As shown in **Figure 3C**, in the molecular function, the majority of shared DEGs were joined in cytokine activity, receptor-ligand activity, chemokine activity, immune receptor activity, etc. It can be observed from **Figure 3D** that in the pathway enrichment, the main shared DEGs were involved in Toll-like receptor signaling pathway, chemokine signaling pathway, IL-17 signaling pathway, viral protein interaction with cytokine and cytokine receptor, etc.

**Table 2:** The top 10 items of genes enrichment of the shared DEGs.

Category	Description	Count	P value
GO BP	GO:0043588 skin development	32	4.08839E-13
GO BP	GO:0008544 epidermis development	33	1.79E-12
GO BP	GO:0030216 keratinocyte differentiation	25	2.97E-11
GO BP	GO:0009913 epidermal cell differentiation	26	2.43E-10
GO BP	GO:0031424 keratinization	19	4.35E-09
GO BP	GO:0030595 leukocyte chemotaxis	18	4.17E-08
GO BP	GO:0032651 regulation of interleukin-1 beta production	12	5.00E-08
GO BP	GO:0071674 mononuclear cell migration	12	5.00E-08
GO BP	GO:0002573 myeloid leukocyte differentiation	17	5.38E-08
GO BP	GO:0002548 monocyte chemotaxis	10	1.09E-07
GO CC	GO:0001533 cornified envelope	8	4.60E-07
GO CC	GO:0034774 secretory granule lumen	19	9.79E-07
GO CC	GO:0060205 cytoplasmic vesicle lumen	19	1.18E-06
GO CC	GO:0031983 vesicle lumen	19	1.29E-06
GO CC	GO:0045095 keratin filament	9	1.95E-05
GO CC	GO:0009897 external side of plasma membrane	18	0.000123568
GO CC	GO:0101002 ficolin-1-rich granule	9	0.000158105
GO CC	GO:1904813 ficolin-1-rich granule lumen	9	0.000158105
GO CC	GO:0005882 intermediate filament	12	0.000167914
GO CC	GO:0045111 intermediate filament cytoskeleton	13	0.000190355
GO MF	GO:0005125 cytokine activity	17	3.81E-07
GO MF	GO:0048018 receptor ligand activity	23	6.37E-06
GO MF	GO:0030546 signaling receptor activator activity	23	7.52E-06
GO MF	GO:0008009 chemokine activity	7	1.45E-05
GO MF	GO:0002020 protease binding	11	1.68E-05
GO MF	GO:0140375 immune receptor activity	10	8.07E-05
GO MF	GO:0048020 CCR chemokine receptor binding	6	0.00011503
GO MF	GO:0042379 chemokine receptor binding	7	0.00015055
GO MF	GO:0004875 complement receptor activity	3	0.000869415
GO MF	GO:0016810 hydrolase activity, acting on carbon-nitrogen (but not peptide) bonds	8	0.000884219
KEGG pathway	hsa04061 Viral protein interaction with cytokine and cytokine receptor	12	3.30E-07
KEGG pathway	hsa05205 Proteoglycans in cancer	15	6.71E-06
KEGG pathway	hsa04060 Cytokine-cytokine receptor interaction	16	0.00013151
KEGG pathway	hsa05132 Salmonella infection	14	0.000243067
KEGG pathway	hsa04620 Toll-like receptor signaling pathway	8	0.000741709
KEGG pathway	hsa04657 IL-17 signaling pathway	7	0.001918461
KEGG pathway	hsa05150 Staphylococcus aureus infection	7	0.002165232
KEGG pathway	hsa04621 NOD-like receptor signaling pathway	10	0.002451624
KEGG pathway	hsa04062 Chemokine signaling pathway	10	0.003336712
KEGG pathway	hsa05202 Transcriptional misregulation in cancer	10	0.003463257

### Identification of the disease-related key module

**Figure 4:**
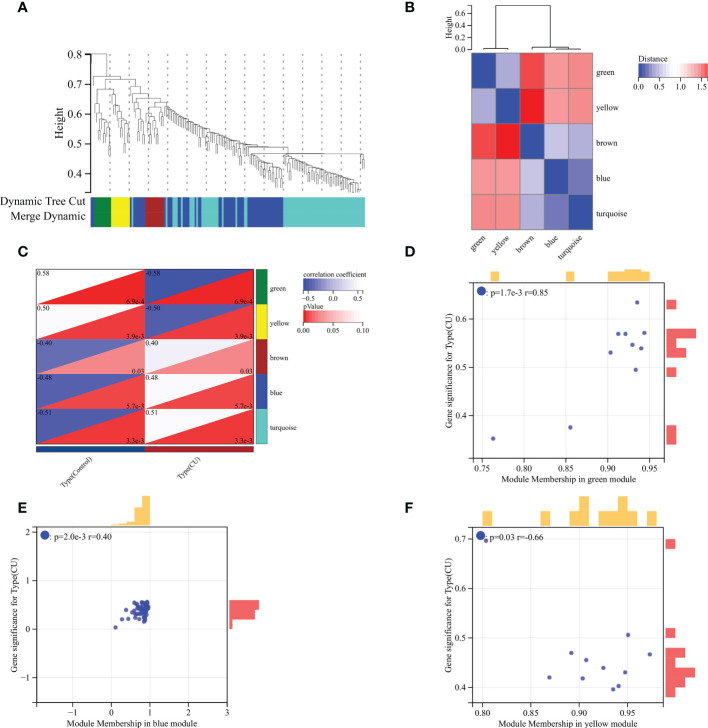
WGCNA analysis. **(A)** Gene clustering dendrogram. **(B)** Heatmap of the association among modules. **(C)** Module-trait relationships heatmap. **(D)** Correlation chart between gene members of the green module and gene significance. **(E)** Correlation chart between gene members of the blue module and gene significance. **(F)** Correlation chart between gene members of the yellow module and gene significance.

The WGCNA was utilized to recognize the most correlated module in CU. There were five gene modules (brown, blue, turquoise, green and yellow modules) depicted by the dynamic tree cut algorithm (**Figure 4A**), and their associations are shown in **Figure 4B**. The brown, blue, and turquoise modules seemed to be positively correlated with CU, and the green and yellow modules seemed to be negatively correlated with it (**Figure 4C**). The association between the color module and gene significance was discerned *via* in-depth calculation. The correlation between green module and gene significance was 0.85 (P-value = 1.7E-3) (**Figure 4D**), the blue module was 0.40 (P-value = 2.0E-3) (**Figure 4E**), and the yellow module was 0.66 (P-value = 0.03) (**Figure 4F**). Therefore, the green, blue and yellow modules were identified as the key modules (78 module genes in **Table S2**).

### PPI network and hub genes analyses

**Figure 5:**
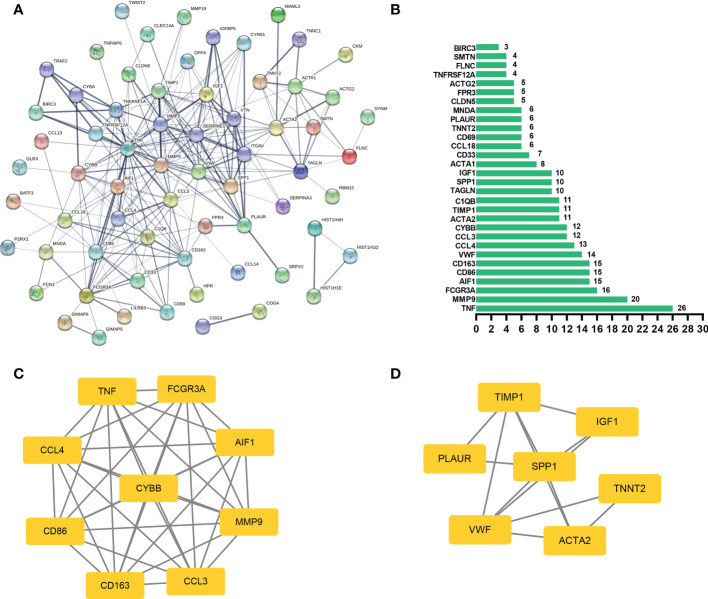
PPI network analysis. **(A)** The PPI network of genes of the key modules identified *via* WGCNA, and the bigger sizes of edges mean the higher degree. **(B)** The top 30 genes of the most connexity in the PPI network. **(C)** The first module of the PPI network. **(D)** The second module of the PPI network.

**Figure 6:**
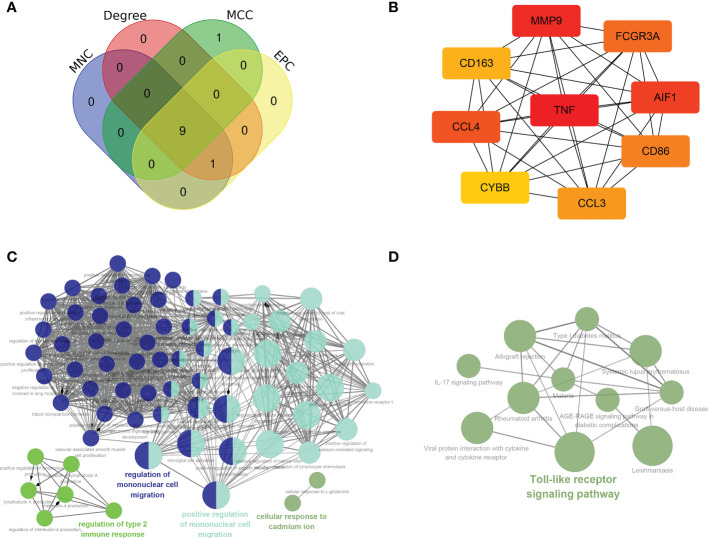
Hub genes analyses. **(A, B)** 9 common hub genes were identified by four algorithms of cytoHubba plug-in. **(C)** The biological process of hub genes *via* the ClueGO. **(D)** The KEGG of hub genes *via* the ClueGO.

In general, genes are not isolated, and the proteins they encode could interact with each other. In order to explore the interaction relationship of proteins, we built a PPI network of genes of the key modules identified *via* WGCNA according to the STRING tool. There were a total of 54 nodes and 168 edges in the network (**Figure 5A**). Moreover, the top 30 genes of the most connexity in PPI network are displayed in **Figure 5B**. The top 2 modules were selected. One had nine nodes and 36 edges (**Figure 5C**), whereas another had seven nodes and 13 edges (**Figure 5D**). We screened the top 10 hub genes by four algorithms of cytoHubba plug-in. Through integrated bioinformatics analysis, 9 common hub genes were identified, containing CD86, FCGR3A, AIF1, CD163, CCL4, TNF, CYBB, MMP9, and CCL3 (**Figures 6A, B** and **Table 3**). And then, we further explored the roles of the hub genes by the plug-in of Cytoscape. As shown in **Figure 6C**, they remained to be involved in immunomodulation containing regulation of mononuclear cell migration, regulation of type 2 immune response, and positive regulation of mononuclear cell migration. Meanwhile, viral protein interaction with cytokine and cytokine receptor, Toll-like receptor signaling pathway, and IL-17 signaling pathway still were the main pathway that they enriched in (**Figure 6D**).

**Table 3:** The hub genes.

Genes	Description	Degree	MCC	MNC	EPC	LogFC (CU)
CD86	CD86 molecule	15	41482	15	19.949	-3.72980534
TNF	tumor necrosis factor	26	37	24	21.332	1.26641463
FCGR3A	Fc fragment of IgG, low affinity IIIa, receptor (CD16a)	16	41476	14	19.768	1.17112479
AIF1	allograft inflammatory factor 1	15	41408	15	19.973	1.02984314
CD163	CD163 molecule	15	41367	14	19.833	1.59973902
CCL4	chemokine (C-C motif) ligand 4	13	41282	13	19.664	2.27644849
CYBB	cytochrome b-245, beta polypeptide	12	41089	11	18.393	1.35127257
MMP9	matrix metallopeptidase 9	20	40978	20	20.892	1.00235674
CCL3	chemokine (C-C motif) ligand 3	12	40704	12	19.863	3.18748669

### Identification of the key genes

**Figure 7:**
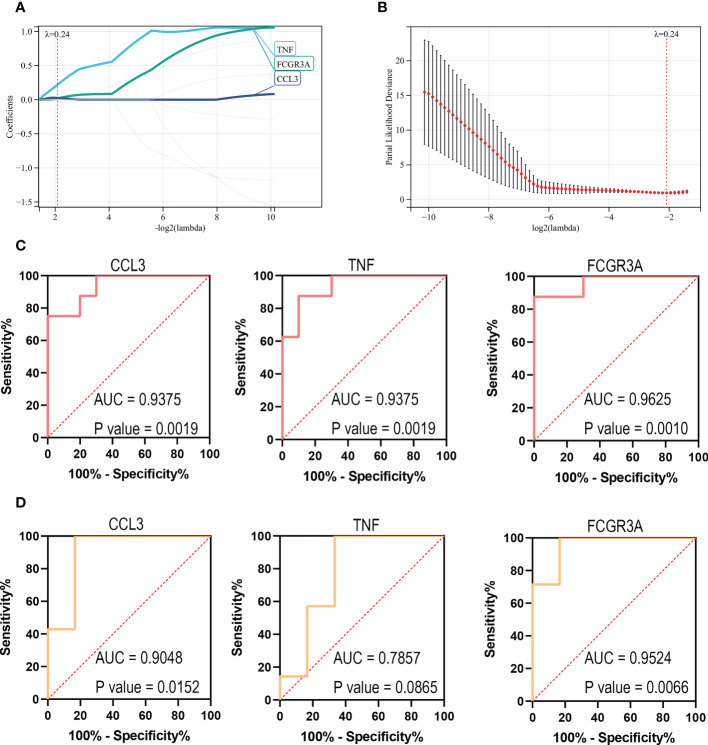
Identification of the key genes. **(A, B)** Further screening of hub genes by machine learning LASSO regression, CCL3, TNF, and FCGR3A, were identified as key genes. **(C)** ROC curves of CCL3, TNF, and FCGR3A in the training set (GSE72540). **(D)** ROC curves of CCL3, TNF, and FCGR3A in the verification set (GSE57178).

In order to further screen reliable biomarkers, we performed the machine learning LASSO regression analysis. The Lambda value was set as 0.235847686271105, and then 3 genes were identified: FCGR3A, CCL3, and TNF. The model formula was as follows: RiskScore=0.21932308885497*TNF+0.0191859986231764*FCGR3A+0.0253400198744963*CCL3 (**Figures 7A, B**). In order to assess the diagnostic specificity and sensitivity of each gene, we established a ROC curve. The results in the train set GSE72540 were as follows: FCGR3A (area under the curve (AUC) 0.9625, P-value =0.0010), TNF (AUC 0.9375, P-value =0.0019) and CCL3 (AUC 0.9375, P-value = 0.0019) (**Figures 7C**); Additionally, they were in validation set GSE57178 as FCGR3A (AUC 0.9524, P-value = 0.0066), TNF (AUC 0.7857, P-value =0.0865) and CCL3 (AUC 0.9048, P-value =0.0152) (**Figures 7D**). These indicated that FCGR3A, TNF, and CCL3 might be the key genes of COVID-19-related CU.

### Immune infiltration analysis

**Figure 8:**
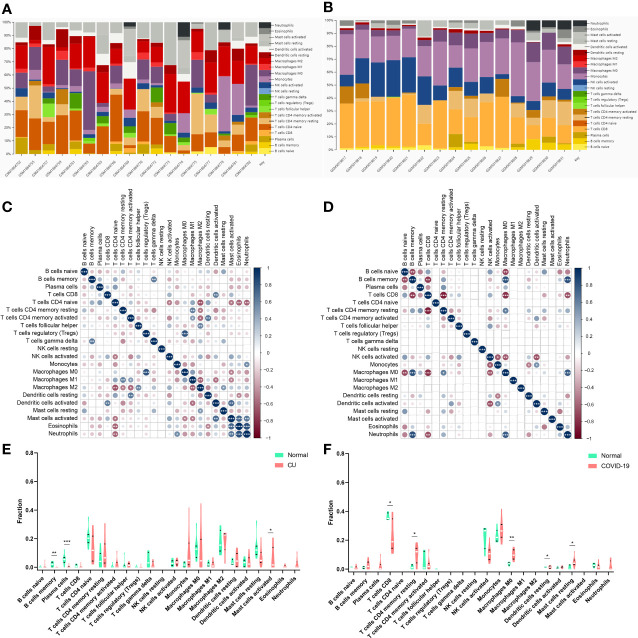
Immune infiltration analysis. **(A)** The ratio of 22 immune cells in CU samples. **(B)** The ratio of 22 immune cells in COVID-19 samples. **(C)** The association among immune cells of CU. **(D)** The association among immune cells of COVID-19. **(E)** The proportion of immune cells in CU and control. **(F)** The proportion of immune cells in COVID-19 and control. *p < 0.05; **p < 0.01; ***p < 0.001.

**Figure 9:**
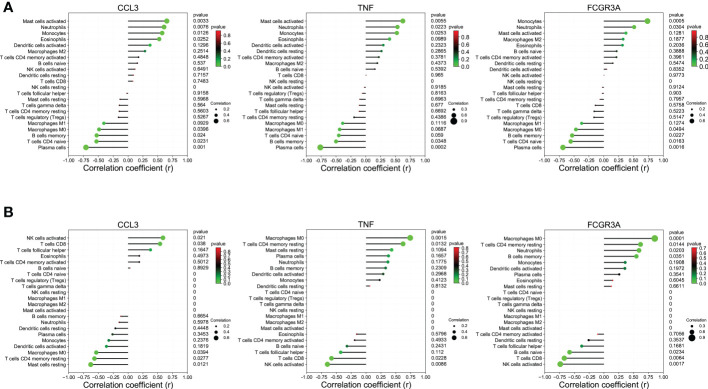
The association between key genes and immune cells. **(A)** CCL3, TNF, and FCGR3A were significantly associated with the primary infiltration cells of CU. **(B)** CCL3, TNF, and FCGR3A were significantly associated with the main infiltration cells of COVID-19.

The CIBERSORT algorithm was applied to investigate the panorama of immune infiltration of CU and COVID-19. The proportion of 22 immune cells of CU and COVID-19 is displayed in **Figures 8A, B**. As shown in **Figure 8C**, relevance analysis between each of the immune cells of CU suggested that mast cells activated were significantly correlated with dendritic cells activated, eosinophils, etc. As shown in **Figure 8D**, the macrophages M0 were significantly related to T cells CD8, B cells memory, B cells naive, etc. in COVID-19. As presented in **Figure 8E**, compared to the control sample, CU displayed a higher proportion of mast cells activated but a lower proportion of plasma cells and B cells memory. Compared to the control sample, COVID-19 had a lower ratio of T cells CD8 but a higher ratio of mast cells resting, T cells CD4 memory resting, macrophages M0, and dendritic cells resting (**Figure 8F**). In addition, Pearson’s correlation coefficient was applied to reveal the relation between the abundance of the immune cells and key genes. As shown in **Figure 9A**, in CU, CCL3 was statistically positively related to mast cells activated, monocytes and eosinophils but negatively to macrophages M0. And TNF was statistically positively related to mast cells activated and monocytes but negatively to plasma cells. Besides, FCGR3A was statistically positively related to monocytes but negatively to macrophages M0 and plasma cells. As shown in **Figure 9B**, in COVID-19, CCL3 was statistically positively associated with T cells CD8 but negatively with mast cells resting and macrophages M0. TNF was statistically positively related to macrophages M0 but negatively to T cells CD8 and NK cells activated. FCGR3A was statistically positively associated with T cells CD4 memory resting and macrophages M0 but negatively with T cells CD8 and NK cells activated.

### Construction of gene-TF- miRNA-lncRNA network of the key genes

**Figure 10:**
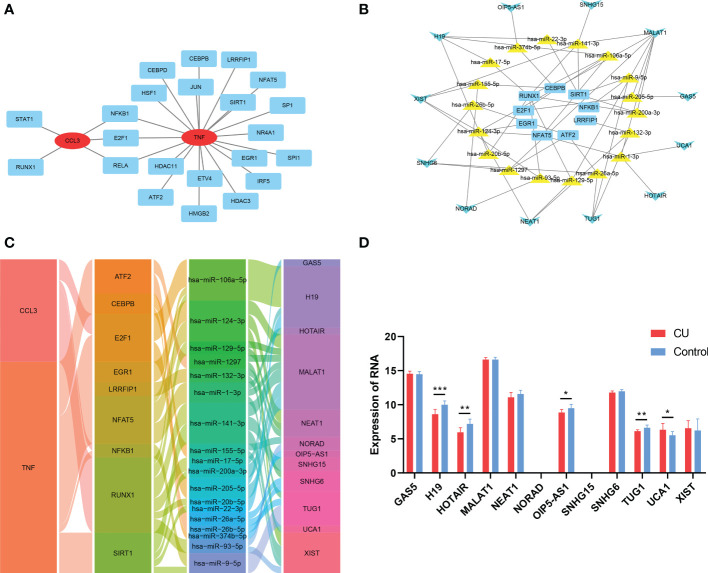
Construction of gene-TF- miRNA-lncRNA network of the key genes. **(A)** The key genes-TFs interaction network. **(B)** The TF-miRNA-lncRNA ceRNA network. **(C)** The key gene-TF-miRNA-lncRNA network. **(D)** The expression of the lncRNAs of the network in CU (GSE57178). *p < 0.05; **p < 0.01; ***p < 0.001.

The TRRUST tool was applied to predict the TFs interacting with the 3 key genes, and TFs-genes regulatory network was visualized *via* Cytoscape and displayed in **Figure 10A**, which contained 22 TFs, 24 nodes, and 25 edges. Subsequently, 6 databases were used to predict the interaction of miRNAs and the 22 TFs. Then 3 databases were used to predict the interaction of lncRNAs and miRNAs targeting the TFs. We explored a TFs- miRNA-lncRNA ceRNA network, containing 39 nodes and 55 edges (**Figure 10B**). Based on integrated bioinformatics analysis, we finally get a key gene-TF- miRNA-lncRNA network including 46 molecular regulation axes such as CCL3/E2F1/hsa-miR-106a-5p/H19, CCL3/E2F1/hsa-miR-106a-5p/H19, CCL3/E2F1/hsa-miR-205-5p/MALAT1, etc (**Figure 10C**). In order to verify the reliability of the network, the expressions of lncRNAs of network in CU (GSE72540) are shown in **Figure 10D**.

### INFLIXIMAB might be the potential treatment drug for COVID-19-related CU

**Figure 11:**
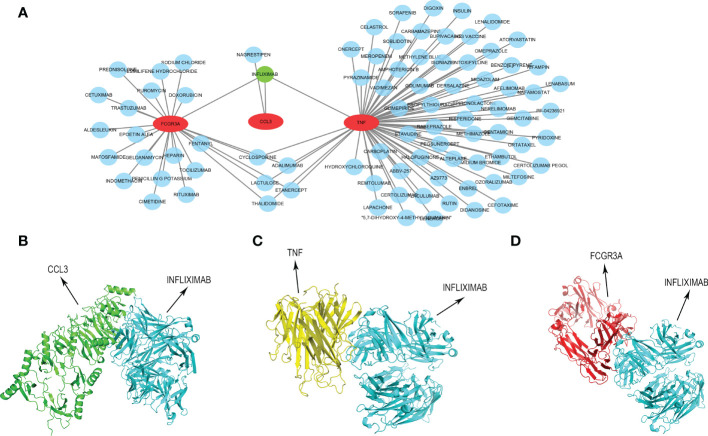
Construction of drug-gene interaction network. **(A)** The interaction between key genes and 87 potential drugs. **(B)** Protein-protein docking of CCL3 and INFLIXIMAB. **(C)** Protein-protein docking of TNF and INFLIXIMAB. **(D)** Protein-protein docking of FCGR3A and INFLIXIMAB.

In order to provide a specific therapeutic drug for COVID-19-related CU, we developed a drug-gene interaction network of the key genes *via* the DGIdb tool. As presented in **Figure 11A** and **Table S3**, 87 different possible drugs were identified, and only INFLIXIMAB can target all key genes simultaneously. Then, we predicted their molecular binding site, and the binding mode of CCL3 with INFLIXIMAB is displayed in **Figure 11B**, TNF with INFLIXIMAB in **Figure 11C**, and FCGR3A with INFLIXIMAB in **Figure 11D**.

## Discussion

Since December 2019, COVID-19, which is caused by SARS-CoV-2, has been spreading worldwide, posing a burden to the global medical system and almost affecting people all over the world (32). In addition to common respiratory symptoms and fever, many COVID-19 patients also behave various rashes (33). Wheal accounts for about 10% of the COVID-19-related skin lesions, and it could develop CU if the course of the disease lasts for more than six weeks (11). And epidemiological studies do report incidence of CU is higher than before the pandemic (2, 34). In addition, CU could be one of the cutaneous adverse reactions following COVID-19 vaccinations (35). The molecular biological mechanisms underlying these, however, remain unknown. Consequently, our research is aimed to describe the possible genetic relation of the two illnesses to further reveal the pathogeny of COVID-19-related CU.

In this study, we identified 1033 DEmRNAs of CU and 6705 DEmRNAs of COVID-19. And then, based on cross analysis, 322 shared DEGs between COVID-19 and CU were identified and executed to function annotation. Our results suggested that the shared DEGs were mainly enriched in skin development (e.g. epidermal cell differentiation and keratinocyte differentiation) and immune cell regulation, especially innate immune cells regulation (such as leukocyte chemotaxis, and monocyte chemotaxis, etc.). In addition, the shared DEGs were mostly enriched in immune-related signaling pathways according to KEGG, such as Toll-like receptor signaling pathway, cytokine receptor, IL-17 signaling pathway, and viral protein interaction with cytokine. Most of the above results were commonly accepted compositional elements of the development of CU and COVID-19 (36, 37). The research reported that monocytes and their subsets are the key sensors of and responders to Toll-like receptor-mediated inflammation, especially after viral infection. Apart from that, classical monocytes may play a central role in severe COVID-19 (38). In addition, the researchers found that in CU, Toll-like receptors agonists could induce the expression of TFs of monocytes, and the TFs increased vascular permeability in a histamine-independent way, leading to the formation of a wheal (39). In some way, these results may hint that the shared DEGs could play a role in the immunoregulation of CU and COVID-19.

Through WGCNA analysis, we recognized three disease-related key modules (green, blue, and yellow). ​Further, based on PPI analysis, 9 hub genes were identified from the key module genes, including CD86, CD163, FCGR3A, AIF1, TNF, CYBB, MMP9, CCL4, and CCL3. They are the familiar immunoregulatory cytokines, but it has not been reported that most of them could link CU with COVID-19. Thus, this might be a new finding. The result of another genes enrichment analysis indicated that the hub genes still were involved in immune cell regulation, containing positive regulation of mononuclear cell migration, regulation of type 2 immune response, etc. Moreover, the primary signaling pathway they enriched in still were viral protein interaction with cytokine and Toll-like receptor signaling pathway. That is to say, the hub genes we identified can be representative of the shared DEGs. In order to improve and simplify the efficacy of prediction and diagnosis of CU, especially COVID-19-related CU, we identified three key genes (FCGR3A, CCL3, and TNF) from hub genes by the machine learning LASSO regression analysis. The reliability of their diagnostic value was verified by ROC curve analysis, suggesting that they could be potential diagnostic biomarkers for COVID-19-related CU.

In order to further understand the immune dysregulation of COVID-19-related CU, we performed the immune infiltration. Many researchers emphasized that mast cell degranulation played a crucial part in the development of CU (40). And it draws increasing attention that monocytes and monocyte-derived cells (macrophages and dendritic cells) are involved in the immunopathology of COVID-19 and may play essential roles in determining disease severity (41). These were further confirmed in our study. We found that compared with the healthy control sample, CU had a higher proportion of mast cells activated, as well as COVID-19 had a higher ratio of macrophages M0, dendritic cells resting, and mast cells resting. Furthermore, mast cells activated were significantly correlated with dendritic cells activated in the CU. Therefore, it could be observed that the high proportion of central immune cells (like mast cells), and the statistical correlation among them, might be a novel perspective for the cellular basis of the development of COVID-19-related CU. Our research further displayed that all the key genes (CCL3, TNF, and FCGR3A) were statistically related to central infiltration cells (e.g. mast cells and macrophages M0) in CU and COVID-19. TNF and CCL3 are famous cytokines and broadly take part in regulating many immune cells and developing inflammatory and allergic diseases. According to the research, TNF and CCL3 could mediate the inhibition of human intestinal mast cell activation by resveratrol (42). Additionally, CCL3 and TNF were found to increase in critically ill patients with COVID-19 and related to increased morbidity and mortality (43). Moreover, the researchers found that FCGR3A is mainly expressed on immune cells (like NK cells) and also correlated with the severity of COVID-19 (44). Therefore, summing up the above, it would be concluded that the key genes (FCGR3A, CCL3 and TNF) we identified could be potential immunomodulation pivots for COVID-19-related CU.

Furthermore, to more systematically understand the modulatory interaction of molecules of COVID-19-related CU, we constructed a key gene-TF-miRNA-lncRNA network. There were 46 regulation axes in the network like CCL3/E2F1/hsa-miR-106a-5p/H19, CCL3/E2F1/hsa-miR-205-5p/MALAT1, CCL3/E2F1/hsa-miR-106a-5p/H19, etc. Studies indicated that although the non-coding RNA could not encode proteins, it was found to participate in regulating the pathogenesis of many skin diseases containing CU (45). Most lncRNAs of the network were proved to be a significant expression in CU, making the network more reliable. Hence, our result may be helpful to understand the molecular mechanism of COVID-19-related CU. Finally, we further identified 87 potential therapeutic drugs for key genes, and there was only one drug, INFLIXIMAB, that could target all key genes. INFLIXIMAB is a drug of great research value. It has been verified to apply for the treatment of immune diseases like ulcerative colitis by several international drug agencies, and the phase II clinical study that patients with severe COVID-19 were treated with INFLIXIMAB was also in progress (46, 47). Therefore, INFLIXIMAB could also be a potential treatment strategy for COVID-19-related CU, and protein-protein docking displayed the exact binding mode, making it more realizable.

However, there was a limitation in our study, which the identified biomarkers were only verified at a theoretical level. But the following experiments *in vivo* and *in vitro* will be the focus of our study in the future.

## Conclusion

Through bioinformatic means, FCGR3A, TNF, and CCL3 were identified as the key genes. In COVID-19-related CU, they are mainly involved in the immunomodulation function, significantly associated with the central infiltration cells, and have statistical diagnostic value, indicating that they might be potential biomarkers for COVID-19-related CU. Overall, our research might provide new ideas for further mechanistic studies.

## Data availability statement

The original contributions presented in the study are included in the article/**Supplementary Material**. Further inquiries can be directed to the corresponding authors.

## Author contributions

SXP and XYZ designed the study. TZ, HF, and SXP performed the data analysis. TZ drafted the manuscript. All authors contributed to the article and approved the submitted version.

## Conflict of interest

The authors declare that the research was conducted in the absence of any commercial or financial relationships that could be construed as a potential conflict of interest.

## Publisher’s note

All claims expressed in this article are solely those of the authors and do not necessarily represent those of their affiliated organizations, or those of the publisher, the editors and the reviewers. Any product that may be evaluated in this article, or claim that may be made by its manufacturer, is not guaranteed or endorsed by the publisher.
