# Supplementary material for: Integrated bioinformatics to identify potential key biomarkers for COVID-19-related chronic urticaria
Source: Front Immunol. 2022 Dec 1;13:1054445. doi: 10.3389/fimmu.2022.1054445 (PMC9751185; doi:10.3389/fimmu.2022.1054445)
Supplement: Supplementary file 1 [file Table_1.doc]

Table S1 The shared DEGs of CU and COVID-19

| Gene symbol (n=322) |
| --- |

ABCB1

ABI3BP

ACACB

ACER1

ACTA1

ACTA2

ACTG2

ADAD2

ADAMTS10

AHNAK2

AIF1

ALOX12B

ANKRD36

ANXA9

APOBEC3A

APOBEC3B

AQP3

AQP5

ARG1

ARL17A

ARL17B

ASB2

ASPRV1

ATF3

ATP12A

AVEN

BATF3

BCAT1

BCL2A1

BCL3

BIRC3

BLMH

BPIFC

BRD3

BTBD16

C10orf99

C1orf115

C1orf35

C1orf68

C1QB

C1QTNF9

C5AR1

CARD18

CCL13

CCL14

CCL18

CCL3

CCL4

CCL8

CD163

CD1A

CD207

CD33

CD69

CD86

CDKN1C

CENPP

CENPT

CH25H

CHAC2

CKM

CLC

CLDN5

CLEC14A

CLEC2D

CMTM5

CNFN

CNTNAP3

COG4

CPA4

CRB3

CRISPLD2

CSF3

CSRNP1

CSTA

CTNNBIP1

CXCR1

CXCR2

CYBB

CYCS

CYP4F22

CYR61

DMKN

DNASE1L2

DNTTIP2

DOK2

DPP4

DUSP1

DUSP2

EBF3

EBNA1BP2

EFHD1

EGFL7

EGR1

ENHO

EPPK1

ERRFI1

ETS2

FAAH2

FABP5

FCGBP

FCGR1B

FCGR3A

FCN1

FERMT1

FFAR2

FGFBP1

FLNC

FNDC4

FOSB

FOSL1

FPGT-TNNI3K

FPR2

FPR3

GIMAP5

GIMAP6

GIMAP7

GJB4

GLRX

GPR183

GSDMA

HAL

HAPLN2

HAS1

HBEGF

HCK

HDC

HERC2

HIST1H1D

HIST1H1E

HIST1H4H

HIST2H2AB

HK2

HLA-DQA2

HLA-DQB2

HOXB2

HOXC8

HPR

HPSE

HSP90AA1

HSPB2

IFI16

IFI30

IGF1

IGF2

IGFBP7

IL20

IL22RA1

ING5

ITM2A

KCNJ15

KCNJ8

KCNK5

KLF10

KLK5

KLK6

KLK8

KRT10

KRT2

KRT6C

KRTAP19-5

KRTAP4-12

KRTAP4-2

KRTAP4-5

KRTAP4-7

KRTAP5-2

KRTAP5-4

KRTAP5-5

LCE1A

LCE3E

LCE5A

LCN2

LHX6

LILRB3

LILRB4

LIMS2

LMO2

LMOD1

LRG1

LRRC2

LRRC59

LY6G6C

LYAR

LYG2

LYNX1

MAL

MAML3

MAP2K3

MAP3K4

MAP3K8

MED8

MEF2C

METRNL

MFAP1

MMP19

MMP7

MMP9

MNDA

MSL1

MSMB

MT1H

MT2A

MTHFD1L

MTHFD2

MYC

NAMPT

NEURL1B

NMU

NOP16

NR4A2

NR4A3

NRXN2

NUAK1

OGG1

OLFML2A

OSMR

OTX1

P2RX1

PCDH19

PCP4

PDPN

PIK3IP1

PLAUR

PLCB4

PLEKHM1

PLXND1

PNO1

PNP

POPDC2

PPIF

PRDM1

PRSS22

PSG8

PTGDS

PYDC1

RBM15

RDH12

RGS16

RGS20

RIOK2

RPA4

SAA2

SALL2

SAMD4A

SCN4B

SERPINA3

SERPINB13

SERPINE1

SLAMF1

SLC20A1

SLC22A7

SLC46A2

SLC5A1

SLC5A6

SLC8A1

SLITRK6

SLPI

SLURP1

SMARCA4

SMO

SMPD3

SMTN

SNAI1

SPARCL1

SPEG

SPEN

SPP1

SPRR1B

SPRR2E

SPRR2G

SRM

SRPX2

ST6GALNAC1

STEAP4

STOX2

SYNE2

SYNM

SYT17

TAGLN

TBX1

TBX2

TBX3

TCF4

TCF7L1

TEAD4

TESC

TET1

TEX101

TGM1

THBD

THG1L

TIMP1

TIMP3

TLR4

TNF

TNFAIP6

TNFRSF10C

TNFRSF12A

TNFRSF25

TNKS1BP1

TNNT2

TRIB1

TSPAN7

TTC21B

TUBA4A

TUBB2A

TWIST2

UAP1

UTS2R

VNN2

VPS18

VSTM1

VWF

WNT6

XDH

YOD1

ZADH2

ZBED6

ZC3H12A

ZNF462

ZNF486

ZNF593

|  |
| --- |
