# Supplementary material for: Integrated bioinformatics to identify potential key biomarkers for COVID-19-related chronic urticaria
Source: Front Immunol. 2022 Dec 1;13:1054445. doi: 10.3389/fimmu.2022.1054445 (PMC9751185; doi:10.3389/fimmu.2022.1054445)
Supplement: Supplementary file 2 [file Table_2.doc]

Table S2 The genes of key modules

| Genes | Modules |
| --- | --- |
| ACTA1 | yellow |
| ACTA2 | yellow |
| ACTG2 | yellow |
| ASB2 | yellow |
| CKM | yellow |
| FLNC | yellow |
| P2RX1 | yellow |
| PCP4 | yellow |
| SMTN | yellow |
| SYNM | yellow |
| TAGLN | yellow |
| ABI3BP | blue |
| AIF1 | blue |
| ANKRD36 | blue |
| BATF3 | blue |
| BIRC3 | blue |
| C1QB | blue |
| CCL13 | blue |
| CCL18 | blue |
| CCL3 | blue |
| CCL4 | blue |
| CD163 | blue |
| CD33 | blue |
| CD69 | blue |
| CD86 | blue |
| CENPP | blue |
| COG4 | blue |
| CSRNP1 | blue |
| CYBB | blue |
| CYR61 | blue |
| DPP4 | blue |
| FCGR3A | blue |
| FCN1 | blue |
| FERMT1 | blue |
| FPR3 | blue |
| GLRX | blue |
| HAPLN2 | blue |
| HDC | blue |
| HIST1H1D | blue |
| HIST1H1E | blue |
| HIST1H4H | blue |
| HPR | blue |
| KRT6C | blue |
| LILRB3 | blue |
| MAML3 | blue |
| MMP19 | blue |
| MMP9 | blue |
| MNDA | blue |
| NR4A3 | blue |
| NUAK1 | blue |
| PLAUR | blue |
| RBM15 | blue |
| SERPINA3 | blue |
| SPP1 | blue |
| SRPX2 | blue |
| TET1 | blue |
| TIMP1 | blue |
| TNF | blue |
| TNFAIP6 | blue |
| TNFRSF12A | blue |
| TNKS1BP1 | blue |
| TNNT2 | blue |
| TWIST2 | blue |
| UAP1 | blue |
| WNT6 | blue |
| ZBED6 | blue |
| ZNF462 | blue |
| ZNF486 | blue |
| CCL14 | green |
| CLDN5 | green |
| CLEC14A | green |
| EBF3 | green |
| GIMAP5 | green |
| GIMAP6 | green |
| IGF1 | green |
| SCN4B | green |
| TSPAN7 | green |
| VWF | green |
