# Supplementary material for: Integrated bioinformatics to identify potential key biomarkers for COVID-19-related chronic urticaria
Source: Front Immunol. 2022 Dec 1;13:1054445. doi: 10.3389/fimmu.2022.1054445 (PMC9751185; doi:10.3389/fimmu.2022.1054445)
Supplement: Supplementary file 3 [file Table_3.doc]

|  |
| --- |

| Table S3 Drug-gene interaction network | |  |
| --- | --- | --- |
| Genes | Drugs | Interaction Score |
| CCL3 | INFLIXIMAB | 3.09 |
| CCL3 | NAGRESTIPEN | 30.91 |
| FCGR3A | SODIUM CHLORIDE | 0.37 |
| FCGR3A | EPOETIN ALFA | 0.27 |
| FCGR3A | ETANERCEPT | 0.95 |
| FCGR3A | RITUXIMAB | 1.66 |
| FCGR3A | CETUXIMAB | 0.54 |
| FCGR3A | ADALIMUMAB | 0.76 |
| FCGR3A | INFLIXIMAB | 0.64 |
| FCGR3A | TRASTUZUMAB | 0.74 |
| FCGR3A | PENICILLIN G POTASSIUM | 1.03 |
| FCGR3A | TOCILIZUMAB | 1.29 |
| FCGR3A | PREDNISOLONE | 0.21 |
| FCGR3A | CYCLOSPORINE | 0.08 |
| FCGR3A | HEPARIN | 0.18 |
| FCGR3A | TESMILIFENE HYDROCHLORIDE | 5.15 |
| FCGR3A | GELDANAMYCIN | 0.37 |
| FCGR3A | LACTULOSE | 1.29 |
| FCGR3A | CIMETIDINE | 0.52 |
| FCGR3A | THALIDOMIDE | 0.26 |
| FCGR3A | PUROMYCIN | 0.64 |
| FCGR3A | DOXORUBICIN | 0.05 |
| FCGR3A | FENTANYL | 0.21 |
| FCGR3A | MAFOSFAMIDE | 1.29 |
| FCGR3A | INDOMETHACIN | 0.15 |
| FCGR3A | ALDESLEUKIN | 0.3 |
| TNF | CARBAMAZEPINE | 0.08 |
| TNF | BUPIVACAINE | 0.18 |
| TNF | HALOFUGINONE | 0.3 |
| TNF | ETANERCEPT | 1.24 |
| TNF | ADALIMUMAB | 1.39 |
| TNF | INFLIXIMAB | 1.55 |
| TNF | ALTEPLASE | 0.1 |
| TNF | INSULIN | 0.05 |
| TNF | CERTOLIZUMAB PEGOL | 0.91 |
| TNF | GOLIMUMAB | 4.55 |
| TNF | RABEPRAZOLE | 0.15 |
| TNF | 5,7-DIHYDROXY-4-METHYLCOUMARIN | 0.23 |
| TNF | MILTEFOSINE | 0.36 |
| TNF | MEROPENEM | 1.82 |
| TNF | SORAFENIB | 0.02 |
| TNF | CARBOPLATIN | 0.02 |
| TNF | PYRIDOXINE | 0.61 |
| TNF | BENZO[E]PYRENE | 0.3 |
| TNF | SPIRONOLACTONE | 0.1 |
| TNF | DIDANOSINE | 0.15 |
| TNF | GLIMEPIRIDE | 0.23 |
| TNF | ATORVASTATIN | 0.03 |
| TNF | OMEPRAZOLE | 0.06 |
| TNF | METHIMAZOLE | 0.1 |
| TNF | PROPYLTHIOURACIL | 0.11 |
| TNF | LAPACHONE | 0.07 |
| TNF | HYDROXYCHLOROQUINE | 0.17 |
| TNF | CYCLOSPORINE | 0.03 |
| TNF | CEFOTAXIME | 0.45 |
| TNF | OZORALIZUMAB | 1.82 |
| TNF | PEGSUNERCEPT | 1.82 |
| TNF | DIGOXIN | 0.03 |
| TNF | DERSALAZINE | 0.23 |
| TNF | ONERCEPT | 0.91 |
| TNF | LENERCEPT | 0.91 |
| TNF | NERELIMOMAB | 0.91 |
| TNF | PLACULUMAB | 2.73 |
| TNF | AFELIMOMAB | 1.82 |
| TNF | AZ9773 | 0.91 |
| TNF | PF-04236921 | 0.45 |
| TNF | RUTIN | 0.18 |
| TNF | AMPHOTERICIN B | 0.13 |
| TNF | NAFAMOSTAT | 0.3 |
| TNF | HOMIDIUM BROMIDE | 0.03 |
| TNF | LACTULOSE | 0.45 |
| TNF | CELASTROL | 0.23 |
| TNF | GENTAMICIN | 0.09 |
| TNF | RIFAMPIN | 0.1 |
| TNF | ORTATAXEL | 0.23 |
| TNF | SOBLIDOTIN | 0.91 |
| TNF | CERTOLIZUMAB | 0.91 |
| TNF | ABBV-257 | 0.45 |
| TNF | REMTOLUMAB | 0.45 |
| TNF | ETHAMBUTOL | 0.45 |
| TNF | LENABASUM | 0.91 |
| TNF | THALIDOMIDE | 0.26 |
| TNF | METHYLENE BLUE | 0.1 |
| TNF | PYRAZINAMIDE | 0.23 |
| TNF | PENTOXIFYLLINE | 0.03 |
| TNF | ISONIAZID | 0.14 |
| TNF | MIDAZOLAM | 0.05 |
| TNF | VADIMEZAN | 0.18 |
| TNF | LENALIDOMIDE | 0.13 |
| TNF | RISPERIDONE | 0.05 |
| TNF | GEMCITABINE | 0.02 |
| TNF | STAVUDINE | 0.13 |
| TNF | ENBREL | 0.91 |
| TNF | BCG VACCINE | 0.15 |
